# EXISTING STRATEGIES FOR ELECTRONIC DATA COLLECTION BY ELDER ABUSE MULTI-DISCIPLINARY TEAMS

**DOI:** 10.1093/geroni/igz038.1579

**Published:** 2019-11-08

**Authors:** Tony Rosen, David Burnes, Darin Kirchin, Alyssa Elman, Risa Breckman, Mark Lachs

**Affiliations:** 1 Weill Cornell Medical College / NewYork-Presbyterian Hospital, New York, New York, United States; 2 University of Toronto, Toronto, Ontario, Canada; 3 Weill Cornell Medical College, New York, New York, United States

## Abstract

Elder abuse cases often require integrated responses from social services, medicine, civil legal, and criminal justice. Multi-disciplinary teams (MDTs), which meet periodically to discuss and coordinate interventions for complex cases, have developed in many communities. Little is known about how these MDTs collect case-level data. Our objective was to describe existing strategies of case-level electronic data collection conducted by MDTs across the United States as a preliminary step in developing a comprehensive database strategy. To identify MDTs currently collecting data electronically, we used a snowball sampling approach discussing with national leaders. We also sent an e-mail to the National Center for Elder Abuse listserv inviting participation. We identified and reviewed 11 databases from MDTs. Strategies for and comprehensiveness of data collection varied widely. Databases used ranged from a simple spreadsheet to a customized Microsoft Access database to large databases designed and managed by a third-party vendor. Total data fields collected ranged from 12-338. Types of data included intake/baseline case/client information, case tracking/follow-up, and case closure/outcomes. Information tracked by many MDTs, such as type of mistreatment, was not captured in a single standard fashion. Documentation about data entry processes varied from absent to detailed. We concluded that MDTs currently use widely varied strategies to track data electronically and are not capturing data in a standardized fashion. Many MDTs collect only minimal data. Based on this, we have developed recommendations for a minimum data set and optimal data structure. If widely adopted, this would potentially improve ability to conduct large-scale comparative research.

